# Bridging the Gap: Optimizing OPAT Transitions to Skilled Nursing Facilities

**DOI:** 10.1093/ofid/ofag136

**Published:** 2026-03-14

**Authors:** Meera Mehta, Carissa Tedeschi, Jack Zhang, Sejal Morjaria, Jessica Johnson

**Affiliations:** Department of Pharmaceutical Services, West Virginia University Hospitals, Morgantown, West Virginia, USA; Department of Pharmaceutical Services, West Virginia University Hospitals, Morgantown, West Virginia, USA; Department of Pharmaceutical Services, West Virginia University Hospitals, Morgantown, West Virginia, USA; Department of Medicine, Section of Infectious Diseases, West Virginia University, Morgantown, West Virginia, USA; Department of Medicine, Section of Infectious Diseases, West Virginia University, Morgantown, West Virginia, USA

**Keywords:** OPAT, skilled nursing facility, care transitions, discharge errors, medication reconciliation

## Abstract

We evaluated patients discharged to skilled nursing facilities for outpatient parenteral antimicrobial therapy (OPAT). Among 89 patients, discharge medication errors and OPAT-related interventions were common, including laboratory monitoring, therapeutic adjustments, and adverse event management; 32 patients (36%) experienced at least 1 documented discharge medication error, with a total of 44 errors identified across the cohort. These findings highlight transition-of-care vulnerabilities and underscore the importance of structured OPAT oversight to improve patient safety.

Outpatient Parenteral Antimicrobial Therapy (OPAT) has become an essential component of modern infectious diseases (ID) management, offering patients the opportunity to complete intravenous (IV) antimicrobial therapy outside of the acute care hospital setting [1–3]. Outpatient parenteral antimicrobial therapy use has increased due to provider expertise, cost pressures of inpatient care, patient preference for outpatient treatment, and expansion of structured programs [4–7].

As the demand for OPAT continues to grow, a significant number of patients requiring prolonged IV antimicrobial therapy are discharged to skilled nursing facilities (SNFs), rather than home health services [8, 9]. Because SNF patients often have complex medical needs and face unique challenges, OPAT services represent a critical advancement in their care, ensuring continuity of antimicrobial management in an environment where oversight can otherwise be fragmented [10–12]. However, OPAT involvement does not fully eliminate these risks, and transitions to SNFs remain vulnerable to communication gaps, coordination challenges, and medication errors.

Communication gaps, discharge reconciliation errors, cost barriers (eg, daptomycin), and inconsistent lab oversight can compromise outcomes and increase readmission risk [13, 14]. A failure modes and effects analysis within an established OPAT program identified critical vulnerabilities in care transitions, especially in follow-up and communication with SNFs, underscoring the need for targeted improvements in OPAT–SNF coordination [15].

This article examines how the OPAT program supports WVUH patients discharged to SNFs, highlighting care transition challenges and strategies to improve collaboration and outcomes.

## METHODS

The WVUH OPAT program operates through a multidisciplinary team comprised of ID physicians, ID pharmacists, nurses, and an administrative assistant. The OPAT team functions as a formal consult service, with inpatient OPAT consultations initiated via an order in the electronic medical record. The OPAT team then provides recommendations regarding antimicrobial selection, dosing, duration, laboratory monitoring, and postdischarge coordination, with documentation of the plan in the medical record. Upon patient discharge, the team performs an antimicrobial discharge reconciliation to ensure the prescribed plan is communicated to and implemented correctly across all postdischarge care settings. The team then provides weekly lab monitoring, oversees antimicrobial adjustments and conducts patient follow-up to ensure safe treatment completion and early management of complications in the outpatient setting (Supplementary Figure 1).

We conducted a retrospective chart review of WVUH OPAT patients (n = 89) discharged to 56 distinct SNFs within our regional network (n = 89 patients across these SNFs) to assess OPAT-related interventions, discharge medication errors, and barriers to discharge. Patients ≥18 years old who received an inpatient OPAT consult between January 1 and 7 April 2023 were included; patients discharged to a rehabilitation facility or home with home health were excluded. Each patient was included once; no repeat SNF discharges occurred during the study period. Data extracted from the EMR included demographics, Charlson Comorbidity Index (CCI), renal dysfunction, and discharge antimicrobial regimens. Renal dysfunction was defined per CCI criteria as moderate (serum creatinine >3 mg/dL) or severe (dialysis, kidney transplant, or uremia).

Recorded OPAT interventions included lab monitoring, dose or duration changes, antimicrobial escalation or de-escalation, and adverse event management, along with discharge medication errors and cost, clinical, or formulary-related barriers. The primary outcome was the frequency and type of OPAT interventions. Secondary outcomes included discharge medication errors and discharge barriers. When discharge errors or barriers were identified, the OPAT team promptly contacted SNF staff, pharmacists, and prescribers to correct antimicrobial dosing, duration, and monitoring during early transition verification. The team addressed formulary and cost barriers by coordinating alternative regimens with SNFs, pharmacy, and the inpatient team.

Discharge errors were identified through routine OPAT transition-of-care verification. As part of standard workflow, the OPAT team contacted receiving SNFs to confirm antimicrobial agent, dose, duration, and laboratory monitoring orders, and documented any discrepancies or required corrections in the WVUH EMR. We did not have direct access to SNF medical records but communicated routinely with SNF nursing staff to verify orders.

Data were summarized using descriptive statistics, with categorical variables reported as frequencies and percentages and continuous variables as medians with interquartile ranges. All analyses were performed using Microsoft Excel (Microsoft Corporation, Redmond, WA). The data that support the findings of this study are available from the corresponding author upon reasonable request. This study was approved by the Institutional Review Board. Patient consent was not required because it was a retrospective review of existing medical records involving minimal risk to participants, and all data were de-identified prior to analysis. The data underlying this article will be shared on reasonable request to the corresponding author.

## RESULTS

### Patient Characteristics

A total of 89 patients discharged from West Virginia University Hospitals (WVUH) to a SNF for completion of IV antimicrobial therapy were included. The median age was 70 years (IQR, 63–78), and 45 patients were male (51.0%). The cohort had a high comorbidity burden, with a median CCI score of 5 (IQR, 4–6). Additional demographic and clinical characteristics are summarized in Supplementary Table 1.

Daptomycin (37, 41.6%), vancomycin (23, 25.8%), ceftriaxone (18, 20.2%), cefepime (16, 18.0%), and ertapenem (11, 12.4%) were the most prescribed IV antimicrobials at SNF discharge.

Bone and joint infections were the most common indication for therapy (50, 56.2%), followed by skin and soft tissue infections (18, 20.2%) and endovascular infections (15, 16.9%). The full distribution of infections treated is provided in Supplementary Table 1.

### Types of Hospital Discharge Barriers

Of the 89 patients we followed, 9 had a documented barrier to discharge. The most common reason was cost (n = 6), followed by SNF formulary restrictions (n = 2) and clinical considerations (n = 1). The antimicrobial most frequently associated with these barriers was daptomycin (n = 7), which is typically the highest-cost agent used in this population and therefore more prone to insurance denials or facility nonformulary status.

### Discharge Errors

**Figure 1.**
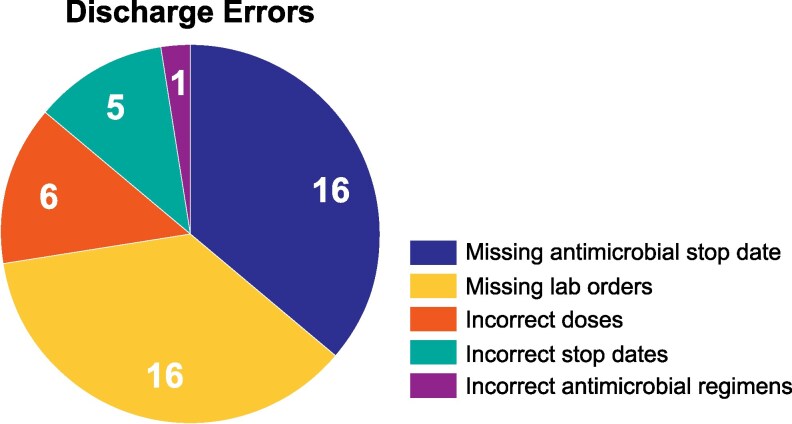
Distribution of discharge errors among 89 patients discharged to skilled nursing facilities for outpatient parenteral antimicrobial therapy.

Among the 89 patients discharged to SNFs, 32 patients (36.0%) experienced at least 1 documented discharge error, with a total of 44 errors identified across the cohort. The most common errors involved incomplete antimicrobial orders, including missing antimicrobial stop dates (16, 36.36%) and absent laboratory monitoring orders (16, 36.36%). Additional errors included incorrect antimicrobial dosing (6, 13.64%), incorrect stop dates (5, 11.36%), and incorrect antimicrobial regimens (1, 2.28%); Figure 1.

### Outpatient Parenteral Antimicrobial Therapy Interventions During Skilled Nursing Facilities Stay

Among documented OPAT interventions during SNF stays, therapeutic plan adjustments and lab monitoring issues were the most common, each accounting for 23 interventions, many of which were related to vancomycin. Adverse event management accounted for 12 interventions and included leukopenia during vancomycin therapy (n = 3) and vancomycin-associated acute kidney injury prompting antimicrobial modification (n = 1). It also included elevated creatinine kinase during daptomycin therapy (n = 4), ceftriaxone-related nausea/vomiting/diarrhea (n = 1), metronidazole-associated dysgeusia (n = 1), daptomycin-associated rash (n = 1), and concern for daptomycin-related eosinophilic pneumonitis (n = 1). A smaller number of interventions were categorized as “other,” including OPAT education and transition-of-care coordination (Table 1).

**Table 1.** Types and Frequency of OPAT-related Interventions Identified During Skilled Nursing Facility Stays

Type of Intervention	Count
Lab monitoring (including vancomycin)	23
Lab monitoring (vancomycin only)	9
Therapeutic plan adjustment	Total = 23 Duration adjustment = 9 Antibiotic adjustment = 1 Dose adjustment = 9 5 vancomycin-related Antibiotic escalation = 2 Antibiotic de-escalation = 2
Adverse event management	Total = 12 4 vancomycin-related
Other	Total = 8 OPAT education = 5 Transition of care = 1 Other recommendations = 2

## DISCUSSION

Effective OPAT coordination between hospitals and SNFs is essential but often hindered by logistical and communication challenges. Studies show that poor discharge communication frequently leads to incomplete or missing information, compromising continuity of care [16].

These documentation errors can directly impact the accuracy of antimicrobial and laboratory orders, sometimes resulting in delays or inappropriate therapy that place patients at risk for complications and contributing to adverse downstream patient outcomes [16, 17]. At WVUH, the OPAT team identified 44 discharge errors among 32 SNF patients (see Figure 1) and made 66 interventions for 43 patients (see Table 1), most often for lab monitoring, adverse events, and dose adjustments—frequently involving vancomycin.

Skilled nursing facilities pose challenges for patients on IV antibiotics, including communication barriers, formulary restrictions, limited resources, and high drug costs (eg, daptomycin). Some facilities cannot support frequently dosed or continuous infusion therapies, increasing the risk of delays and errors. Additionally, variability in SNF willingness to collaborate with OPAT programs further complicates care; some facilities prefer to manage antimicrobial therapy independently, often due to limited awareness of the value added by ID-trained OPAT teams. This reluctance, combined with the difficulty of arranging ID follow-up, can hinder optimal care, particularly when complex regimens or close monitoring are required. Notably, patients themselves report significantly higher satisfaction with OPAT delivered at home compared with SNFs and are more likely to recommend home care to others [1, 3].

Safe SNF transitions for IV antibiotic patients require strong OPAT oversight to prevent errors, delays, and readmissions. To address this, the OPAT team created a 1-page educational summary for SNFs detailing OPAT roles, common errors, and communication pathways, reviewed directly with staff to improve coordination (Supplementary Figure 1). Information from this handout was either presented in person or discussed over the phone with each of the 56 SNFs the OPAT team coordinated care with.

This retrospective, single-center study with a small sample and short timeframe may limit generalizability and introduce inclusion bias based on site-specific practices. Despite these limitations, the findings highlight key vulnerabilities in care transitions and the importance of structured OPAT oversight to improve antimicrobial safety for patients discharged to SNFs.

## Supplementary Material

ofag136_Supplementary_Data

## Supplementary Data

Supplementary materials are available at *Open Forum Infectious Diseases* online. Consisting of data provided by the authors to benefit the reader, the posted materials are not copyedited and are the sole responsibility of the authors, so questions or comments should be addressed to the corresponding author.
