# Supplementary material for: Bridging the Gap: Optimizing OPAT Transitions to Skilled Nursing Facilities
Source: Open Forum Infect Dis. 2026 Mar 14;13(3):ofag136. doi: 10.1093/ofid/ofag136 (PMC13014465; doi:10.1093/ofid/ofag136)
Supplement: ofag136_Supplementary_Data [file ofag136_supplementary_data.zip › Supplemental Table 1 new.pdf]

Supplemental Table 1

Characteristic	Value
Age, years, median (IQR)	70 (63-78)
Male sex - n (%)	45 (51%)
Race	Caucasian 98%<br>African American 2%
**Comorbidities** - n (%)*	
Hypertension	63 (71%)
Coronary artery disease	25 (28%)
Diabetes mellitus (type 2)	22 (25%)
Peripheral vascular disease	5 (6%)
Active malignancy	5 (6%)
Substance use disorder	10 (11%)
HIV/AIDS	2 (2%)
Moderate renal disease†	12 (14%)
Severe renal disease‡	1 (1%)
**Antibiotics used** - n (%)	
Daptomycin	37 (41.6%)
Vancomycin	23 (25.8%)
Ceftriaxone	18 (20.2%)
Cefepime	16 (18.0%)
Ertapenem	11 (12.4%)
Cefazolin	7 (7.9%)
Meropenem	5 (5.6%)
Piperacillin/tazobactam	5 (5.6%)
Ceftazidime	3 (3.4%)
Ampicillin	3 (3.4%)
**Infections treated** - n (%)	
Bone and joint infections	50 (56.2%)
Skin and soft tissue infections (SSTI)	18 (20.2%)
Endocarditis/endovascular infections	15 (16.9%)
Prosthetic joint infections	5 (5.6%)
Urinary tract infections	5 (5.6%)
Bacteremia	3 (3.4%)
Chest infections	2 (2.2%)
Other infections	8 (9.0%)

* Charison Comorbidity Index, mean (SD) 4.2 (1.9)

† Moderate renal disease was defined as chronic kidney disease with serum creatinine >3 mg/dL.

‡ Severe renal disease was defined as dialysis dependence, kidney transplant, or uremia, consistent with Charison Comorbidity Index Criteria.
