# Supplementary material for: Bridging the Gap: Optimizing OPAT Transitions to Skilled Nursing Facilities
Source: Open Forum Infect Dis. 2026 Mar 14;13(3):ofag136. doi: 10.1093/ofid/ofag136 (PMC13014465; doi:10.1093/ofid/ofag136)
Supplement: ofag136_Supplementary_Data [file ofag136_supplementary_data.zip › supplemental figure 1_OFID .pdf]

WVU Medicine

OPAT Program Mission: To ensure that all patients discharged from Ruby Memorial Hospital on intravenous antibiotics complete treatment safely and effectively in the outptient setting

Continuation of Patient Care

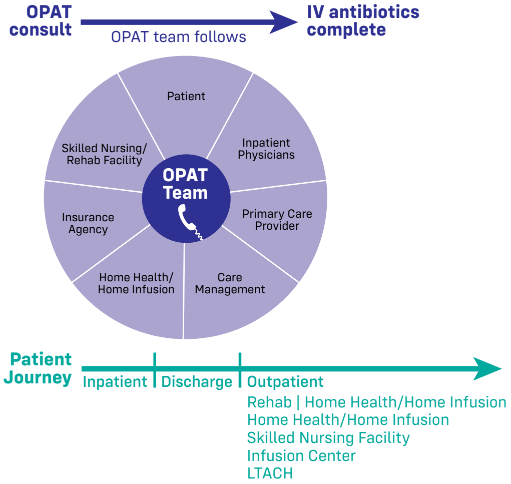

Complications Without OPAT

- Incorrect antibiotic regimen
- Unnecessary IV antibiotics
- Lack of lab monitoring
- Lack of adverse effect management
- Lack of ID follow up
- Discharge without appropriate resources
- Increased readmissions

Benefits of an OPAT Program

- Reduced length of stay
- Cost savings
- Safe transitions of care
- Longitudinal follow up
- Reduced readmissions

Two Rotating ID Physicians

- OPAT Lead Physician
- Consultation to OPAT Pharmacists
- Cosigns Pharmacist Notes
- Educates providers on OPAT program

Five Rotating Pharmacists

- Receive OPAT consults from inpatient services
- Assess patient's clinical status and appropriateness of the proposed OPAT regimen (agent, dose, duration, access)
- Coordinate and communicate recommendations with the ID physician, nursing team, infusion/ dispensing pharmacy, and receiving facility, as applicabale
- Facilitate transition of care and ensure timely follow up as needed
- Monitor and review scheduled labs and adverse effects; recommend therapy adjustments as needed
- Provide patient and caregiver education on antimicrobial therapy and monitoring as needed

Two Rotating Nurses

- Collaborates with home health agencies
- Troubleshoots PICC line issues
- Enters and evaluates outpatient labs
- Makes calls for outpatient labs
- Coodinates antibiotic first doses

OPAT Contact Information

Central OPAT number

- xxx-xxx-4000
- Hours: 8:30 am-5:00 pm, Monday-Friday
- This number can be called for all issues
- Call will be triaged to an available pharmacist

OPAT Weekend On Call

- OPAT Call Hours: Saturday & Sunday 9:00 am - 3:00 pm
- On call number: xxx-xxx-xxxx
